# Oral Nodular Chronic Hyperplastic Candidiasis of the Tongue: A Case Report

**DOI:** 10.7759/cureus.42195

**Published:** 2023-07-20

**Authors:** John Basile, Rania Younis, Reginald Salter, Ronald Brown

**Affiliations:** 1 Department of Oncology and Diagnostic Sciences, University of Maryland School of Dentistry, Baltimore, USA; 2 Division of Restorative Dentistry, Department of Comprehensive Care, Howard University College of Dentistry, Washington, DC, USA; 3 Division of Oral Diagnosis & Radiology, Department of Comprehensive Care, Howard University College of Dentistry, Washington, DC, USA

**Keywords:** immunosuppression, tongue, kid syndrome, candida, nodular chronic hyperplastic candidiasis

## Abstract

Oral nodular chronic hyperplastic candidiasis (CHC) is a rare subset of oral CHC, a relatively uncommon condition associated with immunosuppression. We present a case of a 73-year-old female with nodular CHC of the tongue and a medical history noted for type 2 diabetes. Additionally, we discuss the diagnosis, management, and conditions potentially associated with oral nodular CHC.

## Introduction

Chronic hyperplastic candidiasis (CHC) is a unique clinical subtype of oral candidiasis that presents as whitish oral plaques, usually found at the labial commissure, which cannot be wiped off [1,2]. Other locations for this lesion include the tongue and buccal mucosa [3-5]. CHC can be difficult to diagnose and manage because clinically it can mimic oral neoplasms and is known to be associated with malignant transformation in immunosuppressed patients [2,6,7]. Biopsy specimens may demonstrate invasion by *Candida* hyphae but it is not known if the fungal infection is primary or secondary [4]. A biopsy procedure should be considered in most cases as the epithelium can progress from hyperplasia to dysplasia and eventually to malignancy [6,8].

Holmstrup and Axéll [5] proposed an oral candidiasis classification of acute types, i.e., pseudomembranous and erythematous forms, and chronic types, including pseudomembranous, erythematous, plaque-like, and nodular variants. Nodular CHC is noted for the formation of whitish papular nodules that can be seen in conjunction with other disease states such as keratitis-ichthyosis-deafness (KID) syndrome, an inherited condition characterized by corneal, skin, and hearing abnormalities. Nodular type CHC tends to exhibit variations in the thickness of the epithelium, likely related to a hyperplastic response to the *Candida* infection resulting in a raised, nodular appearance that can be influenced by the virulence of the causative fungal species [1,3,5].

Candida is present in the normal oral flora in between 30% and 55% of healthy adults. It is seen roughly equally in males and females, with colonization increasing in frequency with advancing age, and can cause disease under appropriate conditions [4]. The most common *Candida* species found in oral candidiasis infections, including nodular CHC, is *Candida albicans*, a fungus that exists in both hyphal and yeast forms, presumably due to its greater level of pathogenicity and adherence properties. Approximately 80% of oral candidiasis lesions are due to *Candida albicans*. However, there are a number of other species involved in oral yeast infections including *C. dubliniensis*, *C. glabrata*, *C. krusei*, *C. kefyr*, *C. parapsilosis*, *C. stellatoidea*, and *C. tropicalis*. Predisposing local and systemic factors favoring infection include xerostomia, anemia, antibiotic drug therapy, radiation therapy, and immunosuppression, which could be caused by diabetes mellitus, HIV infection, chemotherapy, steroids or other medications, and systemic illnesses like leukemia [5-8]. Here, we present a case of an elderly female with a history of immunosuppression and dry mouth who also had a medical and family history suggestive of an underlying genetic predisposition to nodular CHC, a unique combination of conditions rarely reported in the literature.

## Case presentation

History of present illness

A 73-year-old female presented to the oral medicine clinic in December of 2021 as a referral from her periodontist with a chief complaint of a “white coating” on her tongue, “stinging tongue bumps,” a sore jaw, and a dry mouth. The condition started years prior, which the patient believed was secondary to acid reflux. Oral thrush was considered a possible diagnosis by the referring dentist. The condition resolved for several years on its own but reappeared in 2019. The patient reported that her tongue was very sore and that she lost much of the ability to taste food. Also, she stated that her “tongue looks like a road map” and that salt made her tongue burn. Previous trial therapies with fluconazole and yogurt suggested by her dentist were not successful in resolving the condition. The patient reported that a biopsy was performed in 2010 and the diagnosis was that the condition was “benign.”

Medical history

Her medical history included a number of surgeries to remove growths and lesions. She had a mole removed from her head and growths removed from both the right and left ears. The patient stated that she was hard of hearing, which she believed was related to these surgeries, reporting a loss of hearing in her right ear following the removal of a lesion in 2003. She also had a tumor removed from her left thigh and reported several back surgeries. The patient was taking valsartan, clonidine, and metolazone for hypertension, metformin and ezetimibe for diabetes, pantoprazole and famotidine for gastric reflux, and low-dose aspirin for decreased platelet adhesion. The patient reported no known drug allergies.

Family history

The patient's father was deaf and died of a stroke at the age of 86 years. The patient’s mother was diagnosed with multiple myeloma and died secondary to pneumonia at the age of 67 years. The patient has two sisters in relatively good health (both breast cancer survivors), one brother deceased, and another brother in relatively good health.

Extra-oral and intra-oral exam

**Figure 1:**
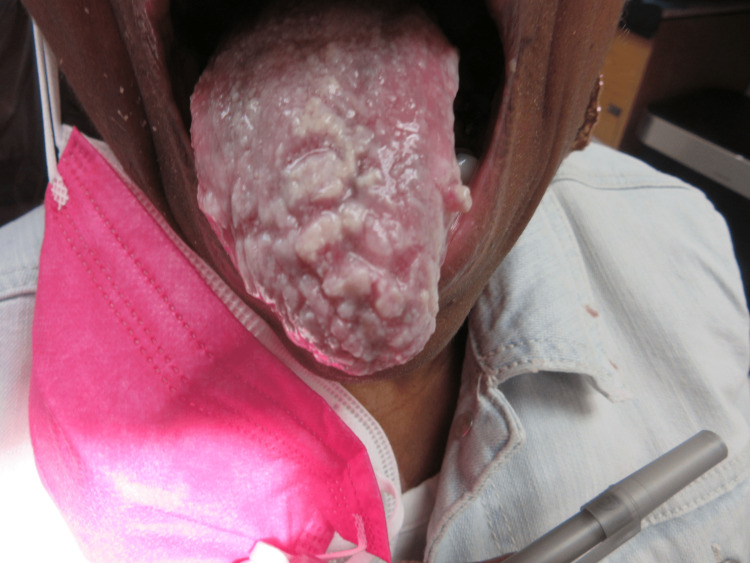
Multiple white papular lesions covering the dorsal aspect of the tongue

Clinically, lymphadenopathy was not observed and the extraoral exam was within normal limits. The tongue was noted for multiple papular white lesions covering the dorsal aspect and a small papular lesion approximately 5 mm in diameter and 7 to 8 mm in length of the middle third of the left lateral border of the tongue that could not be wiped off (Figure 1). Other intra-oral areas were within normal limits. The parotid salivary glands did not demonstrate function bilaterally and the submandibular salivary gland demonstrated only limited function, possibly as a side effect of polypharmacy. The initial differential diagnoses were (1) chronic oral hyperplastic candidiasis, (2) leukoplakia, and (3) human papillomavirus (HPV) infection.

The patient consented to a biopsy procedure. A topical local was applied to a small portion of the affected area and anesthesia (2% lidocaine with 1:100,000 epinephrine) was administered. A 3.5 mm punch biopsy was performed on both the dorsal tongue lesion and the lesion of the lateral left border, and the specimens were sent out to a laboratory for histopathologic evaluation. One week later, the pathology report was received and the patient was called with the results.

**Figure 2:**
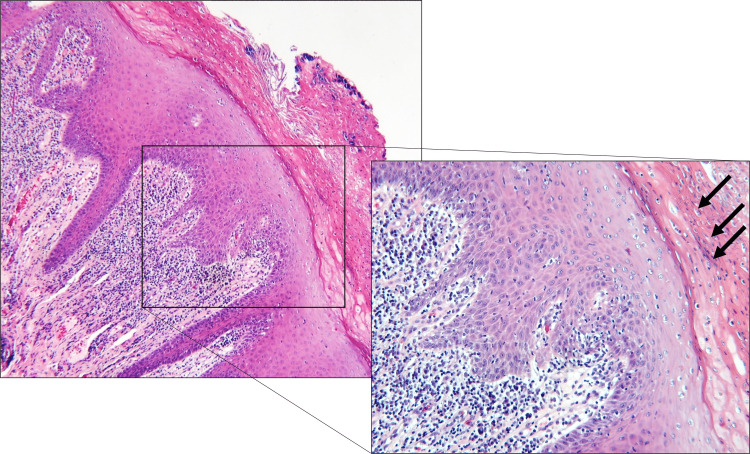
Hematoxylin and eosin-stained section shows stratified squamous epithelium overlying fibrovascular connective tissue There is a robust chronic inflammatory infiltrate at the epithelial-connective tissue interface, while the epithelium exhibits hyperparakeratosis and intraepithelial abscess formation (arrows, inset; original magnification 4X, inset 10X).

**Figure 3:**
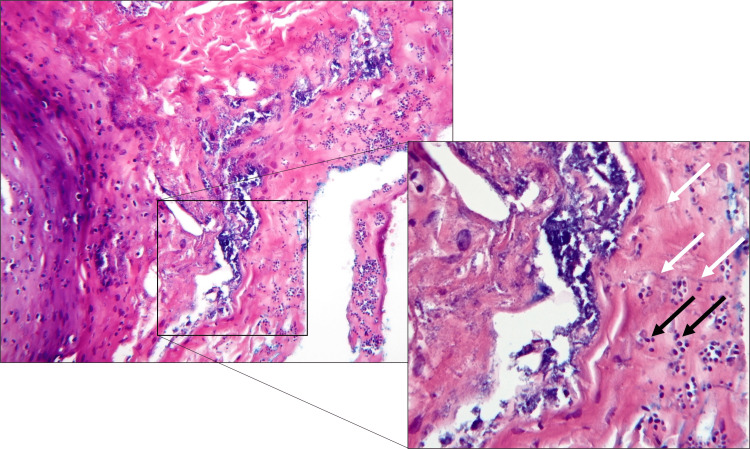
Candida is seen invading the epithelium Hematoxylin and eosin-stained section demonstrating colonization and invasion by both individual yeast cells (black arrows) and hyphae (white arrows, inset; original magnification 10X, inset 20X).

The histopathologic appearance was noted for hyperparakeratinized epithelium featuring intra-epithelial abscesses of lymphocytes (Pautrier's microabscesses) and polymorphonuclear leukocytes (Munro’s microabscesses), a robust chronic inflammatory infiltrate, and isolated lymphoid follicles immediately subjacent to the epithelium. The periodic acid-Schiff stain was positive for numerous mycotic hyphae that were observed invading the epithelium. These histopathologic findings were consistent with nodular CHC (Figures 2, 3).

The results were discussed with the patient and a prescription was ordered for a regimen of 100 mg fluconazole tablets, two to be taken immediately and then one daily for two weeks with three refills. In early January 2022, the patient reported by telephone that after four weeks of fluconazole drug therapy, the tongue lesions and oral pain were resolved. Also, a dry mouth strategy was discussed, including sipping water throughout the day, using various dry mouth products such as Biotene rinse and dry mouth toothpaste, and using a water humidifier in the bedroom to resist drying of the mucosa while asleep.

**Figure 4:**
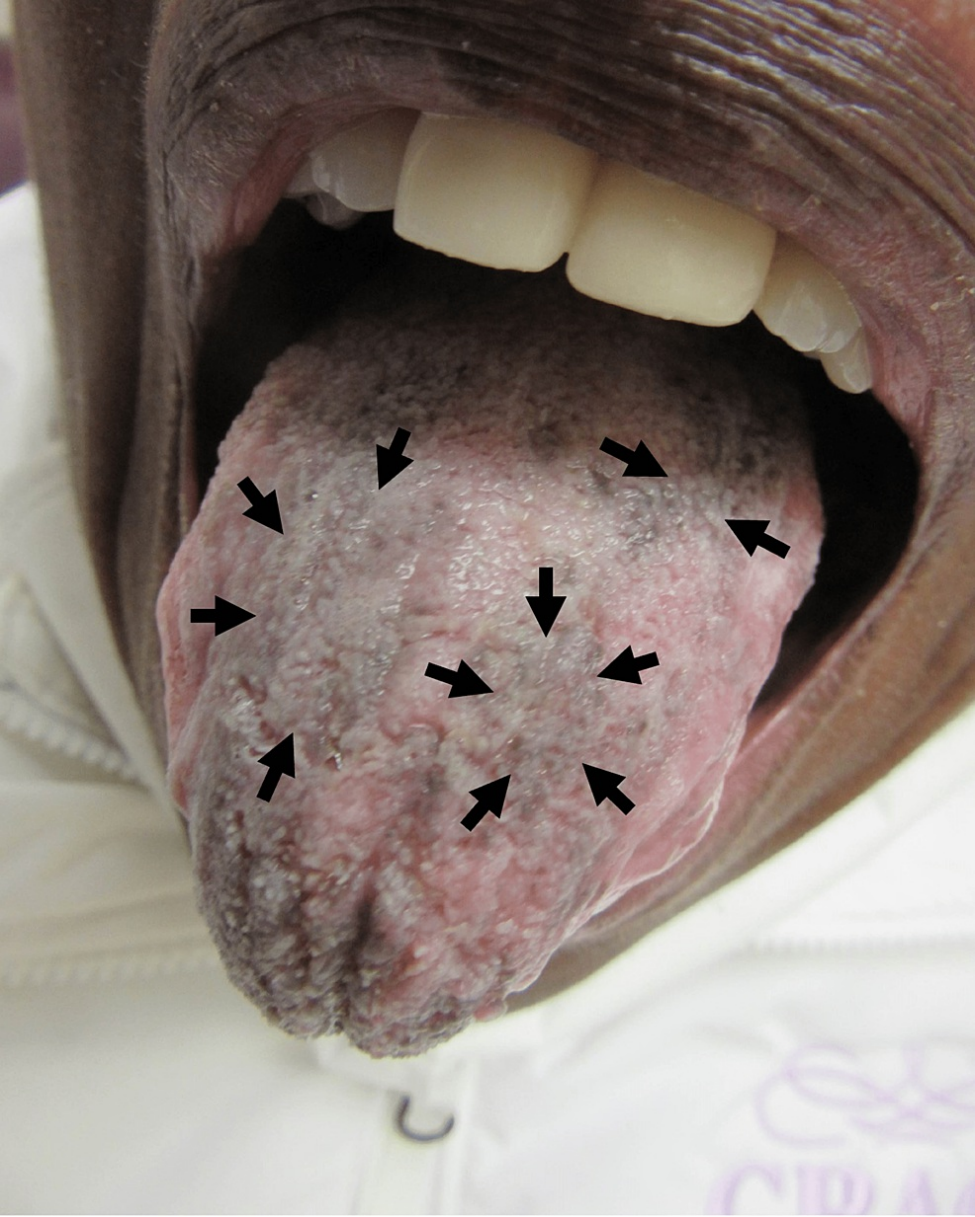
Tongue, post-treatment The appearance of the tongue demonstrating dark staining (black arrows) after the resolution of *Candida* infection.

The patient returned for a follow-up appointment in March 2022. After one month of treatment, the tongue lesions had resolved, although dark staining was noted (Figure 4, black arrows), while the patient’s dry mouth symptoms improved.

At a one-year follow-up, the patient reported that her condition remained stable and that her oral dryness condition was much improved. Considering her medical history and that of her father, the issue of KID syndrome was discussed and it was suggested she should consider undergoing an evaluation with a geneticist. The patient said that she might consider such an evaluation at a later date but was presently not interested. Also, the patient was informed that due to the nature of KID syndrome and nodular CHC lesions with respect to malignant transformation, regular clinical appointments were necessary to monitor both skin and oral mucosa.

## Discussion

Zhang et al. [6], in a study of 48 oral CHC patients, noted a male gender prevalence of approximately three to two and an average age of diagnosis of approximately 55 years. They reported that 21% of CHC patients had varying amounts of epithelial dysplasia with two eventually transforming into a malignancy. They also noted greater therapeutic success with fluconazole drug therapy compared to nystatin. Their conclusion was that the diagnosis and management of CHC was difficult due to its polymorphic clinical presentation and malignant transformation potential. The opinions of Zhang et al. [6] were supported by Lorenzo-Pouso et al. [7], who reported a malignant transformation rate of 12.1% in CHC patients.

Pina et al. [1] reported 36 cases with a diagnosis of nodular CHC. Initially, they considered a large differential diagnosis, including granular cell tumor, verruciform xanthoma, oral condylomata, and verrucous hyperplasia, requiring a thorough histopathologic evaluation to rule in or out any of these possibilities. They further enlarged the differential diagnoses by including neuropathic diagnostic entities such as neurofibroma, schwannoma, perineuroma, solitary circumscribed neuroma, and traumatic neuroma. Later still, they included lymphoproliferative disorders, paracoccidiomycosis, syphilis, and amyloidosis.

With regard to the clinical evaluation of the present case, our differential diagnoses included CHC, leukoplakia, oral squamous cell carcinoma, and HPV infection. After receiving the histopathologic evaluation with a diagnosis of nodular CHC, a two-week regimen of fluconazole was prescribed along with increased hydration and suggestions for over-the-counter dry mouth products.

The histopathologic appearance of CHC is notable for hyperparakeratinized epithelium, a chronic inflammatory infiltrate exhibiting isolated lymphoid follicles subjacent to the epithelium, and intraepithelial abscesses of lymphocytes, polymorphonuclear leucocytes, and associated scattered cellular debris. In our case, the periodic acid-Schiff stain was positive for numerous mycotic hyphae that demonstrated invasion of the epithelium.

In the study by Pina et al. [1], they reported successful results by treating lesions surgically, while in the present case, anti-fungal therapy was sufficient to resolve the condition. The size of the lesion in our case was very large compared to what was described by Pina et al. [1], but in both instances, resolutions were achieved without recurrence. With respect to the present case, the patient reported the unsuccessful utilization of fluconazole previous to her presentation to the oral medicine clinician. This may have been a function of mild immunosuppression in the setting of diabetes, as Appleton reported that resistance to antifungal medications may be increased in immunocompromised patients [8]. Furthermore, the virulence of the *Candida* species causing CHC could affect any resistance to therapeutics [1,3,5]. Indeed, a four-week regimen of anti-fungal medication was necessary to resolve the tongue lesions.

KID syndrome is a rare genetic ectodermal condition with relatively few published cases, first reported by Burns [9] in 1915 as a hereditary keratoderma condition with ocular and mucosal involvement. KID syndrome exhibits a clinical triad of progressive vascularizing keratitis, hearing loss, and epithelial manifestations [10-12]. The term “KID syndrome” was coined by Skinner et al. [13] in 1981, and identified such features as deafness, erythrokeratoderma, alopecia, hyperkeratosis of the palms and soles, ophthalmologic defects, and vascularizing keratitis. KID syndrome is associated with tumors as well as viral, fungal, and bacterial infections. The latter findings of infection are significant for our patient, particularly in combination with a medical and family history suggesting a syndrome that is associated with malignancies such as squamous cell carcinoma. Calderon-Castrat et al. [12] reported that the etiology of KID syndrome is secondary to mutations in connexin 26, a gap junction protein responsible for the formation of intercellular channels that also has been implicated in the development of malignancies. Both autosomal dominant and autosomal recessive forms have been identified [10-13].

The medical history of the patient and her father noted hearing loss and undefined skin lesions, a finding that was suspicious for KID syndrome. Therefore, an evaluation of the patient for this condition was attempted. Unfortunately, the patient was not willing to go through genetic testing. Arguing against KID syndrome was the lack of a number of associated findings and symptoms, including erythrokeratoderma, alopecia, hyperkeratosis of the palms and soles, ophthalmologic defects, and vascularizing keratitis. In the end, a diagnosis of KID syndrome was not established.

## Conclusions

In conclusion, a case of oral nodular CHC in an elderly female has been presented, a condition that has been associated with the development of squamous cell carcinoma. The diagnosis was confirmed by biopsy, and the oral lesions were resolved with systemic fluconazole drug therapy. A history of immunosuppression is associated with CHC. Our patient had a medical history noted for type 2 diabetes, which likely predisposed the patient to a candidal infection, as well as being affected by dry mouth. The case was complicated by a history suggestive of a syndrome that would further enhance the patient's risk of developing cancer. Clinicians should become familiar with the appearance of oral nodular CHC along with the associated diagnostic and management concerns. Considering the risk of malignant transformation, early diagnosis and aggressive treatment are critical.
